# Variations in the Availability of Pollen Resources Affect Honey Bee Health

**DOI:** 10.1371/journal.pone.0162818

**Published:** 2016-09-15

**Authors:** Garance Di Pasquale, Cédric Alaux, Yves Le Conte, Jean-François Odoux, Maryline Pioz, Bernard E. Vaissière, Luc P. Belzunces, Axel Decourtye

**Affiliations:** 1 UMT PrADE, Avignon, France; 2 ACTA, Avignon, France; 3 INRA, UR 406 Abeilles et Environnement, Avignon, France; 4 INRA, Unité expérimentale d’entomologie, Le Magneraud, France; 5 ITSAP-Institut de l’abeille, Avignon, France; University of Cologne, GERMANY

## Abstract

Intensive agricultural systems often expose honey bees (*Apis mellifera* L.) to large temporal variations in the availability (quantity, quality and diversity) of nutritional resources. Such nutritional irregularity is expected to affect honey bee health. We therefore tested under laboratory conditions the effect of such variation in pollen availability on honey bee health (survival and nursing physiology—hypopharyngeal gland development and *vitellogenin* expression). We fed honey bees with different diets composed of pollen pellets collected by honey bees in an agricultural landscape of western France. Slight drops (5–10%) in the availability of oilseed rape (*Brassica napus* L.) pollen resulted in significant reductions of all tested variables. Despite some variations in taxonomic diversity and nutritional quality, the pollen mixes harvested over the season had a similar positive influence on honey bee health, except for the one collected in late July that induced poor survival and nursing physiology. This period coincided with the mass-flowering of maize (*Zea mays* L.), an anemophilous crop which produces poor-quality pollen. Therefore, changes in bee health were not connected to variations in pollen diversity but rather to variations in pollen depletion and quality, such as can be encountered in an intensive agricultural system of western France. Finally, even though pollen can be available *ad libitum* during the mass-flowering of some crops (e.g. maize), it can fail to provide bees with diet adequate for their development.

## Introduction

Bee species can be classified into two broad categories regarding their pollen diet: specialists, that feed on a few or even a single plant species, and generalists, that forage on a large array of phylogenetically unrelated plant species [1]. Compared to specialists, generalists usually have a better resilience to environmental changes [2]. Honey bees (*A*. *mellifera* L.), which are extreme generalist, are thus expected to be able to adapt to anthropogenic landscapes, notably in intensive agricultural ones. However, beekeepers have frequently cited starvation and poor foraging conditions as a significant driver of colony losses [3,4]. In addition, Naug [5] suggested that a nutritional stress due to habitat loss plays an important role in honey bee colony losses. The intensification of agriculture often causes a decrease in the diversity of floral resources due to the destruction of natural habitats and the use of monocultures over large areas, but it also affects the quantity of resources available because the flowering period of a crop is usually short [6].

Such changes in the landscape and thus in spatial and temporal availability of foraging resources can influence colonies as it has long been recognized that a lack of food, in particular pollen dearth, contributes to weaken colonies [7,8]. Indeed, pollen nutrients (proteins, lipids, vitamins and minerals) are essential for colony development and survival [9]. Several studies have shown that complete pollen deprivation can impair the longevity [10–13], the development of hypopharyngeal glands (HPGs) (required for the production of brood food) [7,14–17], metabolism [18–21], immunocompetence [17,22], and tolerance threshold of honey bees to pathogens and pesticides [11,17,23–26]. Furthermore, pollen quality and diversity can also affect the size of hypopharyngeal glands and worker longevity [27–30]. A reduction of brood rearing combined with a shorter lifespan of adults can directly impact colony populations [8,31–34]. Pollen resources in an agricultural landscape may be continuously available, but with considerable variations over a season [35–37]. For example, amounts of pollen collected by colonies in an intensive agrosystem in western France follow a very irregular pattern, with a clear depletion period (e.g. 66% decline in pollen harvest) [36,37]. Furthermore, the diversity of pollen species harvested by honey bee colonies shows also considerable temporal variations [6,38,39].

Yet it is not known whether and how the variability of available pollen resources (quantity, quality and diversity) encountered by honey bees in an agricultural landscape influences their health. To improve our knowledge on this question, we first tested under laboratory conditions whether small variation in pollen abundance (as opposed to a total lack of pollen) could significantly affect honey bee health by providing workers with controlled amounts of pollen. Then, by feeding workers with pollen mixes harvested in an intensive agricultural area over different periods, we assessed the influence of diet diversity and quality over time and determined whether or not a particular time period may be critical for honey bees. The impact of the different diets on worker survival and nurse physiology was determined. For that last parameter, we chose to measure the development of HPGs and the level of *vitellogenin* as both are nutritionally regulated via pollen intake and reach a peak at the nurse stage before declining when transitioning to a forager stage [10,40,41]. Nurses secrete 60 to 80% of the brood diet from their HPGs, providing a secretion rich in protein for larvae [42]. *Vitellogenin* is a multifunctional lipoprotein [43] synthetized in the fat body, acting as an antioxidant to promote longevity in both queen and workers [44,45], and it is also used by nurses for the production of brood food [43,46].

## Materials and Methods

### Honey bee rearing and pollen feeding

Experiments were conducted in 2013, in Avignon (France), with colonies of hybrid honey bees (*A*. *m*. *ligustica x A*. *m*. *mellifera*) (no specific permissions were required for these species, locations or activities). One-day-old bees were obtained from three colonies by placing brood combs with late-stage pupae in an incubator at 34°C and 60–70% relative humidity. Bees that emerged within 10 hours were collected, mixed and placed in cages (10.5 cm x 7.5 cm x 11.5 cm) in an incubator at 34°C and 60–70% relative humidity. They were provided *ad libitum* with candy (Apifonda ^®^ + powdered sucrose) and water throughout the experiment. Diets were prepared from bee-collected pollen pellets and supplied to the workers from day 1 to 9. This period of pollen feeding covers the normal pollen-consumption and nursing periods of workers in colonies [10]. Each day, pollen diets were weighed for each cage to determine the amount of pollen consumed per day and per bee. The amount of pollen was adjusted daily to the number of surviving workers (more than 90% during period between day 1 and 9, see the results). Dead bees were counted daily and removed from the cages until the end of the experiment.

#### Influence of a pollen depletion on nurse physiology and survival

To test whether small variations in pollen abundance could significantly affect honey bees, we fed workers with controlled amount of oilseed rape pollen. We determined nurse physiology and survival along a range of decreasing pollen quantities: 100%, 40%, 30%, 20%, 15%, 7% and 0%. The 100% treatment corresponded to the pollen quantity provided daily under the *ad libitum* feeding condition. This quantity was determined with a preliminary experiment, done the week before this experimentation (average of 3.78 mg/worker/day; SD = +/- 0.27 mg; N = 10 cages of 50 bees, data not shown). The amount of pollen consumed by the bees of this 100% treatment amounted to an average of 3.55 mg/worker/day (SD = +/- 0.18 mg, data not shown). To all other treatments, the amounts of pollen provided were fully consumed. Cohorts of 44 one-day old workers were reared in cages and fed with one of the pollen treatment. Each treatment condition was replicated ten times. The pollen of oilseed rape (F_1_ hybrid ‘NK Fair’, Syngenta) was used because of its common occurrence in cereal farmland systems [36]. This pure pollen was collected by colonies on plants grown under two 24 m long x 8 m wide plastic tunnels. The tunnels openings were covered with an insectproof screen to prevent any contamination by pesticides or other pollen types while two honey bee colonies were introduced in the tunnels at the onset of flowering and continuously fitted with bottom pollen traps that were emptied daily. These pollen pellets were stored for one year at—20°C until use and such pollen retains its nutritional value [16].

#### Influence of seasonal diversity and quality of pollen in mixes on nurse physiology and survival

**Fig 1:**
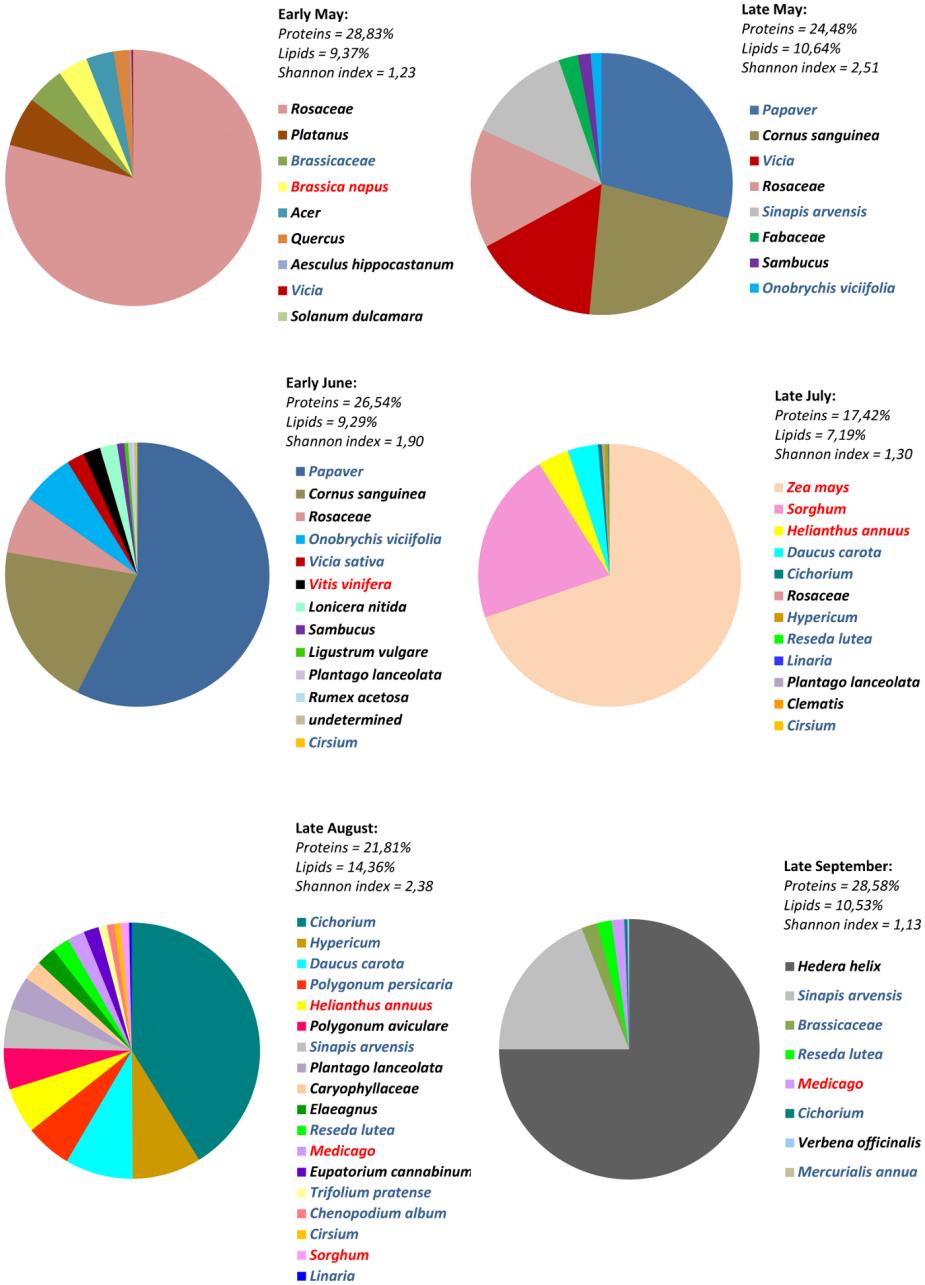
Volume composition and quality of seasonal pollen mixes harvested in an agricultural landscape of western France (46 ° 09′ 13″ N; 0° 41′ 20″ W). Protein and lipid levels, and Shannon Weaver index were calculated for each mix. Pollen protein and lipid are expressed as % of dry matter. Name of species written in red are the main crop species, in blue the fodder species, and in black the wild flowers species.

In this experiment, we tested under laboratory conditions the effects of pollen mixes collected by colonies in an agricultural landscape of western France. The farming landscape was composed of 11% grassland, 26% sunflower (*Helianthus annuus*), 5% oilseed rape, and 4% maize in a 2500 m radius area wise. Fodder crops are permanent or temporary based on legumes or grasses. The different pollen mixes collected over the different seasons were obtained with pollen traps from experiments conducted in 2006 [36] and they were all frozen fresh after harvest. Workers were fed with the 6 pollen mixes made of pollen pellets collected from early May to late September in an apiary located in western France at 46 ° 09′ 13″ N; 0 ° 41′ 20″ W (Fig 1 and S2 Table). Each pollen mix was provided *ad libitum* (20 mg/bee/day, to avoid a bias caused by a possible dearth of pollen) to the workers (10 cages of 47 workers per diet). The protein and lipid contents of the different mixes were determined after the pollen was dried for 24 h at 75°C [35] and the protein and lipid contents were expressed as % of dry matter (Fig 1). The protein content was determined by Kjeldahl analysis (N x 6.25) using a Vapodest 45 (Gerhardt) and according to the procedure ISO 5983–2 norm [47] (INRA-EASM, Surgères, France). After treating pollen with 6 N hydrochloric acid, total lipids were extracted with a chloroform/methanol mixture (2:1, v/v) following the procedure of Folch *et al*. [48]. The presence of pesticide residues in the different pollen diets was checked by chemical analysis (Phytocontrol laboratory, Nîmes, France) performed by gas and liquid chromatographies coupled with mass spectrometry (limit of quantification of 0.01 mg/kg and limit of detection of 0.005 mg/kg) [49]. The samples were analysed for 348 pesticides (S1 Table).

Finally, to estimate the diversity of pollen mixes, we calculated the Shannon Weaver index for each mix, as

H′=−∑pi.log2 pi

where p*i* was the proportion of each pollen type by volume represented in the mixture [50]. The Shannon index increases as the species richness increases, with no upper limits although it is rarely greater than 4 in most ecological studies.

### Physiological analysis

For both experiments, 3 workers by cage were flash frozen in liquid nitrogen at day 10, and their heads and abdomens were separated and stored at– 80°C. Then, the right and left HPGs were dissected on ice in 100 μl of physiological serum (0.9% NaCl w/v) and analysed with an optical microscope linked to a camera (CF 11 DSP, Kappa). The maximum diameter of 15 haphazardly chosen acini by gland was used to determine the development of HPGs (n = 30 acini by bee). This was performed with the Saisam 5.0.1 software (Microvision). The 3 abdomens from the 3 workers by cage were pooled. After RNA isolation, the expression level of *vitellogenin* was determined by quantitative RT-PCR using a StepOne-Plus Real-Time PCR Systems (Applied Biosystems) and the SYBR green detection method and *vitellogenin* expression was normalized to the housekeeping genes *Actin* and e*IF3-S8* [51]. The influence of pollen depletion and seasonal mixes on worker survival was studied on the remaining bees. Dead bees were counted daily and removed from the cages until the last worker died. The bee mortality was followed for 60 days [52], which allowed us to analyse 99.9% of 2870 bees involved in the experiment on the influence of a pollen depletion. But during the following experiment with pollen mixes, mortalities occurred over a longer period, so the duration of observations was increased to 90 days. Survival analysis was then performed on the 98.3% of the 2640 bees used in the experiment on the influence of seasonal diversity and quality of pollen mixes.

### Statistical analysis

Since HPGs sizes were not normally distributed, the effects of pollen diet on this variable were determined using Kruskal-Wallis tests followed by Wilcoxon tests with Bonferroni correction to assess pairwise differences between diet treatments. *Vitellogenin* expression followed a normal distribution, and we used a one-factor ANOVA followed by Tukey post-hoc tests. For analysing the influence of feeding treatments on bee survival, the number of dead bees per day and cage throughout the experiments (60 or 90 days) were transformed in a survival table. A Cox proportional hazards regression model was then used to compare the different treatments, with R functions (coxph) and the package [survival] [53]. Cox models are semi-parametric analyses specifically designed to test the effect of covariates on the time lapse before the occurrence of mortality. Finally, differences in the consumption of seasonal mixes were analysed using a Kruskal-Wallis test followed by Wilcoxon tests with Bonferroni correction. All analyses were performed with the statistical software R [54].

## Results

### Influence of a pollen depletion on nurse physiology and survival

**Fig 2:**
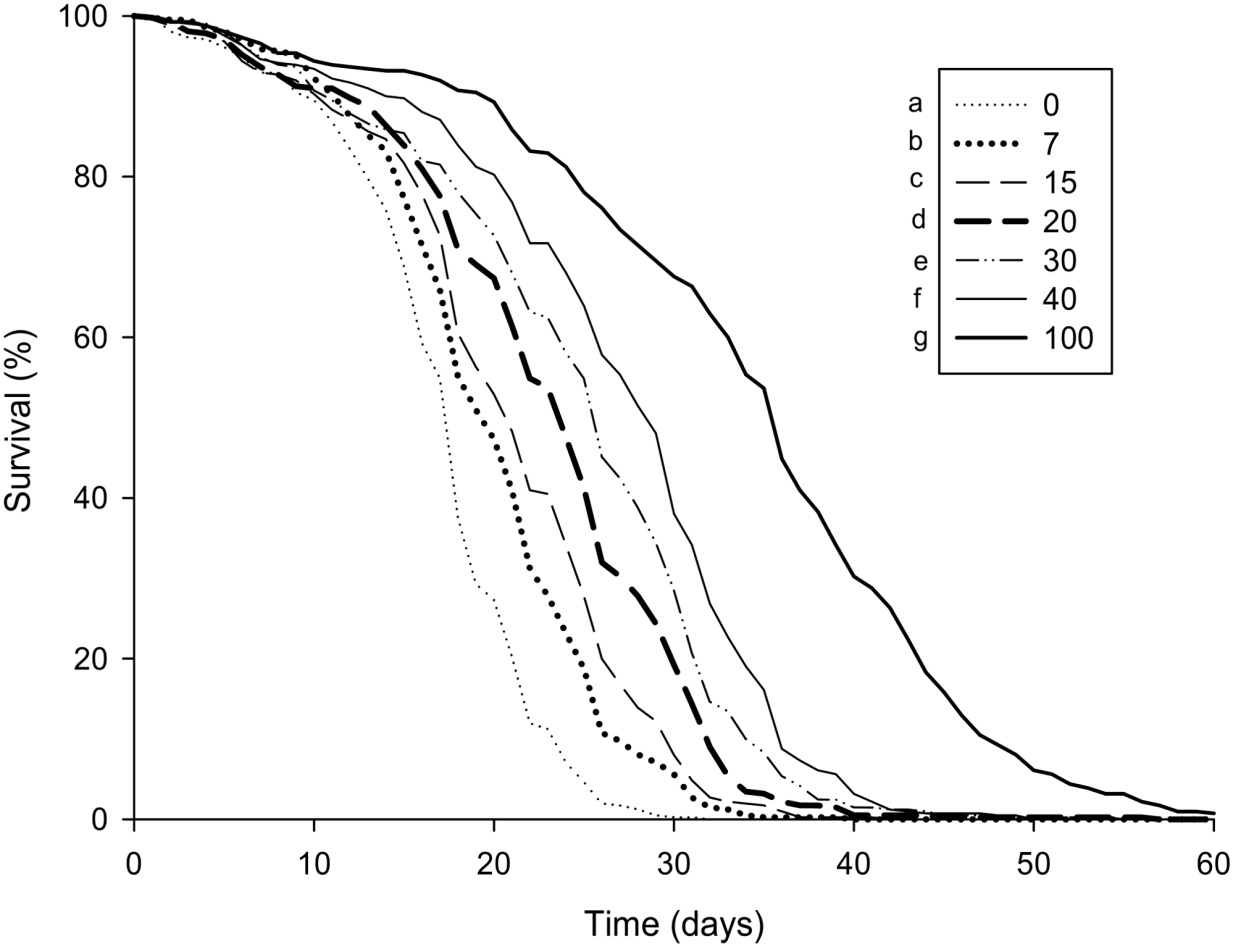
Effect of pollen depletion on worker survival. Data show the percentages of surviving workers over 60 days (n = 10 cohorts per treatment) according to the amount of pollen available (by weight in %). Different letters denote significant differences (p < 0.001, Cox proportional hazards regression model).

Worker survival was highly dependent on the amount of pollen available. The more pollen the honey bees received, the longer they lived, with all quantitative treatments being different from each other (Cox proportional hazards regression model, p < 0.001, Fig 2).

**Fig 3:**
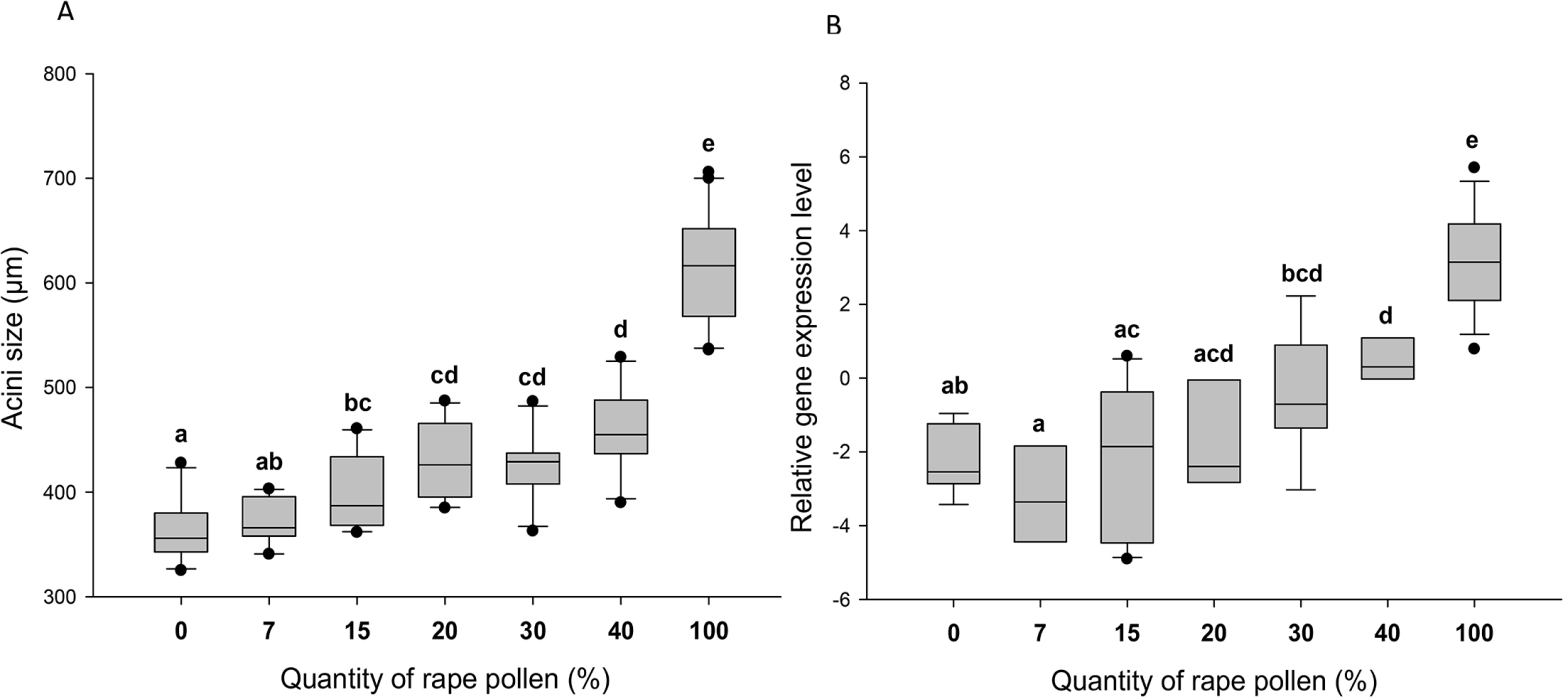
Effects of oilseed rape pollen depletion on (A) the diameter of hypopharyngeal gland acini and (B) *vitellogenin* expression levels, in honey bees. Box plots are shown for 30 workers (acini) and 10 pools of 3 bees (*vitellogenin*) for each diet treatment. Different letters indicate significant differences between pollen quantities (p < 0.001, Wilcoxon tests with Bonferroni correction for HPGs and Tukey post-hoc tests for *vitellogenin* expression levels, respectively). Boxes show 25th and 75th percentiles range with the line denoting the median. Whiskers encompass 90% of the individuals, beyond which outliers are represented by dots.

The size of the HPGs and the expression level of *vitellogenin* were significantly affected by the quantity of oilseed rape pollen consumed (HPGs: Kruskal-Wallis H = 160.70, p < 0.001, Fig 3A; *vitellogenin*: F = 24.51; df = 6, 60; p < 0.001, Fig 3B). The acini size and the level of *vitellogenin* also increased gradually with the amount of available pollen. However, workers that received the lowest amount of pollen (7%) had glands that did not differ in size from those that had a pollen-free diet. Similarly, low quantity of pollens (7 to 30%) did not induce significant changes in *vitellogenin* levels compared to bees that received no pollen at all.

### Influence of seasonal diversity and quality of pollen in mixes on nurse physiology and survival

The levels of proteins and lipids in the different pollen mixes ranged from 17 to 29% and from 7 to 14%, respectively, with the mix of “late July” having the lowest pollen quality (Fig 1). Pollen mixes from “late July” were essentially composed of pollens types from crops as compared with others periods, for which pollen was mainly from wild flowers. The Shannon-Weaver index ranged between 1.13 and 2.51. The diversity of pollen mixes varied among periods, but there was no clear decrease at a given period. The “late July” pollen was not the one with the lowest diversity index. Finally, some pesticide residues (fungicides and herbicides) above the detection limit were found in the pollen mix from different seasonal periods (Table 1). However, no residue was detected in the “late July” mix.

**Table 1:** Pesticide residues in the seasonal pollen mixes.

Pollen sample	Pesticides	Class	Quantity (mg/kg)
Early May	Aclonifen	herbicide	0.032
	Cyprodinil	fungicide	0.012
	Flusilazole	fungicide	0.013
	Metolachlor	herbicide	0.014
Late May	Flusilazole	fungicide	0.015
Early June	Flusilazole	fungicide	< 0.01
	Pyrimethanil	fungicide	< 0.01
Late July	/	/	/
Late August	Trifluraline	herbicide	0.06
Late September	Trifluraline	herbicide	< 0.01

The presence of pesticide residues in the different pollen mixes was assessed with a limit of quantification of 0.01 mg/kg and a limit of detection of 0.005 mg/kg.

**Fig 4:**
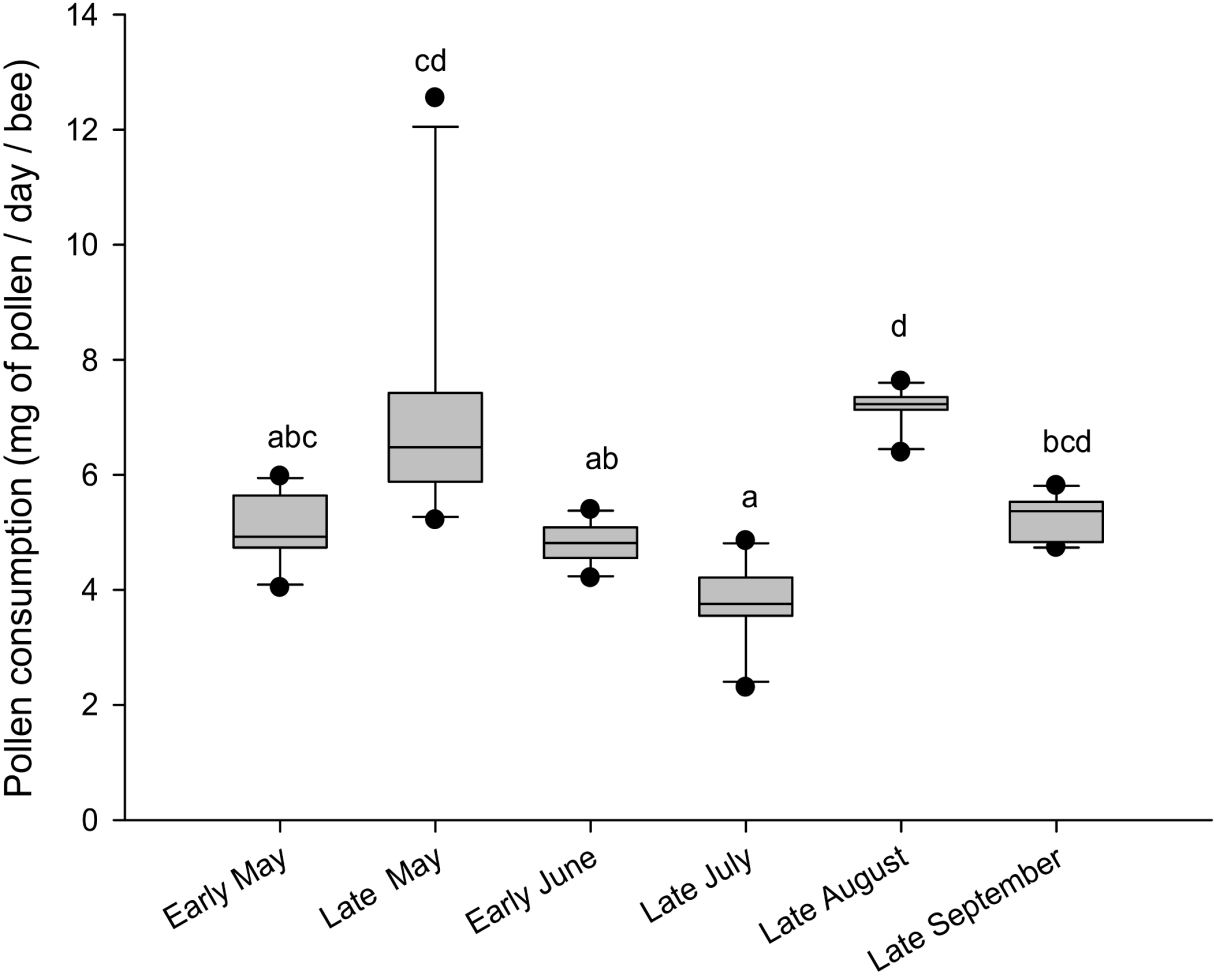
Amounts of the different pollen mixes consumed per bees. Box plots are shown for 10 cages per treatment. Different letters indicate significant differences between the amounts of pollen consumed (p < 0.001, Wilcoxon test with Bonferroni correction). Boxes show 25th and 75th percentiles range with line denoting the median. Whiskers encompass 90% of the individuals, beyond which outliers are represented by dots.

Pollen mixes were provided *ad libitum*, but were not consumed equally by workers (Kruskal-Wallis, H = 46.64, p < 0.001, Fig 4). Notably, the mix from “late July” was poorly consumed (less than 4 mg/bee/day), but its average consumption was not different from the mix of “early May” and “early June”.

**Fig 5:**
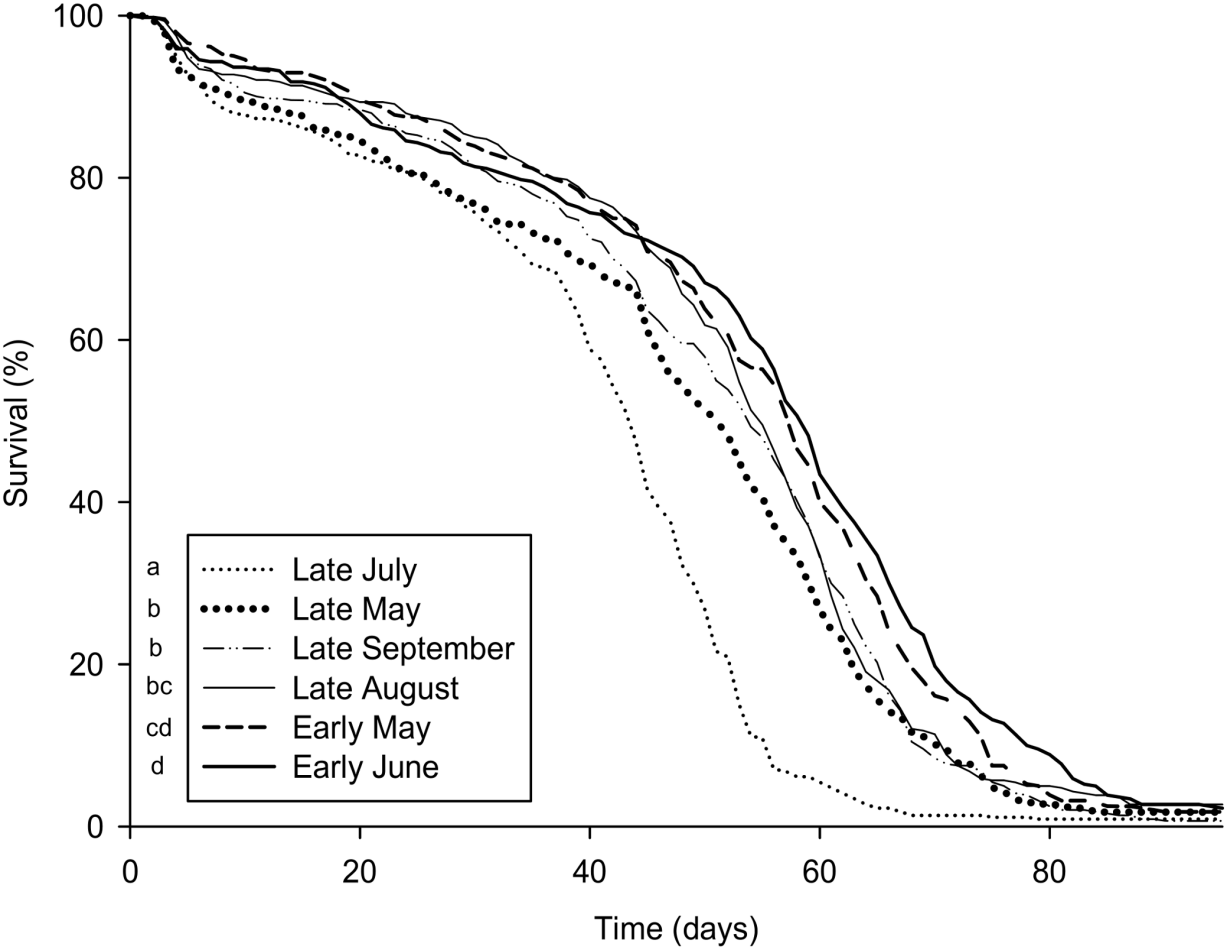
Influence of different seasonal pollen mixes on bee survival. Data show the percentages of surviving workers over 90 days (n = 10 repetitions per treatment). Different letters denote significant differences (p < 0.001, Cox proportional hazards regression model).

**Fig 6:**
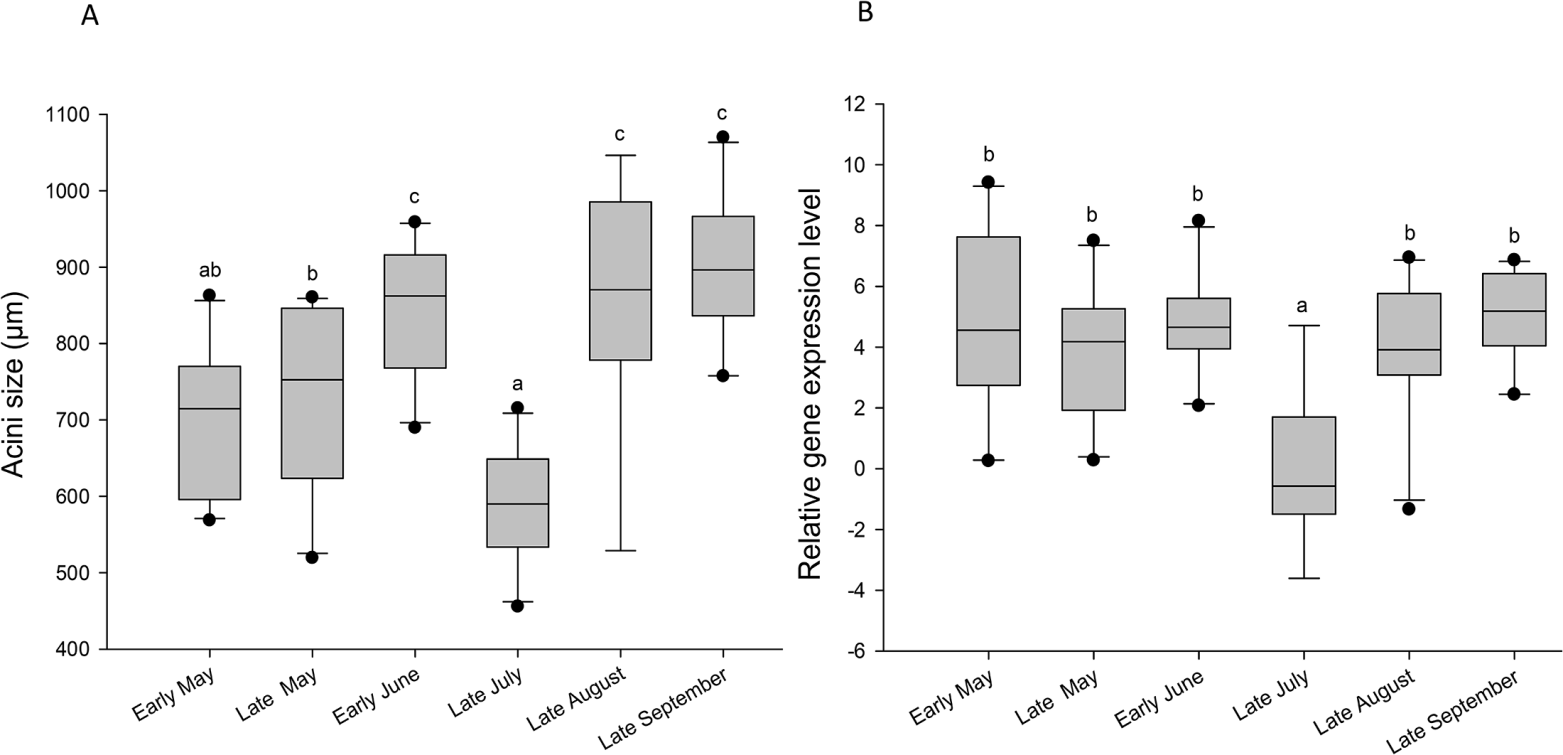
Influence of different seasonal pollen mixes on (A) the diameter of hypopharyngeal gland acini and (B) the *vitellogenin* expression levels, in honey bee workers. Box plots are shown for 30 bees (acini) and 10 pools of 3 bees (*vitellogenin*) per diet treatment. Different letters indicate significant differences between pollen quantities (p < 0.001 based upon Wilcoxon tests with the Bonferroni correction for HPGs and Tukey post-hoc tests for *vitellogenin* expression levels). Boxes show 25th and 75th percentiles range with the line denoting median. Whiskers encompass 90% of the individuals, beyond which outliers are represented by dots.

Most pollen mixes induced slight changes in worker survival but there was no clear link between the species richness of pollen mixes and bee survival (Figs 1 and 5). However, workers provided with the “late July” mix exhibited a dramatic reduction in longevity compared to all others mixes. Similarly, the HPGs development and *vitellogenin* expression were the lowest in the workers fed with the “late July” mix (Kruskal-Wallis, H = 31.35, p < 0.001, Fig 6A and F = 5.01; df = 6, 62; p < 0.001, Fig 6B, respectively). “Early June”, “late August” and “late September” mixes gave the largest acini, but there was no difference in *vitellogenin* expression between the different mixes besides the one of “late July”.

## Discussion

Despite the extreme flexibility of their pollen-foraging behavior and the considerable breadth of their pollen diet, honeybee colonies are often reported as under stress in intensive agricultural landscape [37]. However, the extent to which the modifications of the floral resources affect workers is not easy to analyse. Indeed, there are many other sources of variability beyond resource availability and quality, that can affect workers in the field, such as climate, pests and diseases, and pollutants in general and pesticides in particular [55–59]. Here, by feeding workers under controlled conditions with field-collected pollen pellets diets using a standard protocol, we were able to assess the effects of some components of the nutritional stress associated with an agrosystem on honey bee health.

### Influence of pollen depletion on nurse physiology and survival

Variations in pollen availability are commonly observed in intensive agricultural landscapes between the flowering periods of mass-flowering crops such as oilseed rape and sunflower [37,60]. It is well-established that pollen intake is important for worker survival [10,13,30], but less is known about the impact of small variation in pollen abundance. We found here that slight reductions in pollen availability significantly reduced worker survival under laboratory conditions. As an example, a decrease of pollen consumption of 10% caused an average decrease of 2–3 days in bee longevity, indicating that worker longevity is highly dependent on the amount of available pollen (Fig 2). Nursing physiology was also affected by a gradual reduction in pollen availability. Our results indicate that the rearing capacities of nurse honey bees are impacted by pollen availability because the development of the HPGs is highly dependent on protein intake (Fig 3A) [7,16]. Thus, it might not be surprising to find that the more pollen workers consumed, the more proteins were directed to the acini. Fernandes-da-silva and Zucoloto [61] also tested the effect of pollen on the development of HPGs in the stingless bee *Scaptotrigona depilis Moure* (*Hymenoptera*, *Apidae*). By testing three amounts of pollens (0.25 g, 0.50 g and 0.75 g for 8 days), they found that the two lowest amounts were not sufficient for a good development of the glands. Based on these results, they calculated that at least 0.80 mg pollen/bee/day is necessary to provide sufficient gland development, which was equivalent to the 20% treatment in our tests. But in honey bees, access to only 20% of the full pollen intake resulted in a poor development of the HPGs.

Similarly, the *vitellogenin* expression tended to increase gradually with the amount of pollen available. Bitondi and Simoes [62] tested the effects of 0%, 15% and 50% of pollen diet on *vitellogenin* expression level in Africanized honey bees. They found that workers that received 50% of pollen had more *vitellogenin* than those that received no pollen or only 15% of pollen. This is consistent with our results because the workers that received 15% and 40% of pollen expressed significant differences in *vitellogenin* expression. Altogether, those results show that some honey bee life traits (e.g. longevity, nursing physiology) are highly sensitive to minor changes in pollen availability.

### Influence of seasonal diversity and quality of pollen in mixes on nurse physiology and survival

Pollen diversity has been shown to provide honey bees with a more adequate diet than a monotonous regime [22,27,30]. Therefore, we would expect that beneficial effects on bee health increase as the pollen species richness increases. However, despite important changes in pollen composition of the mixes over the seasons (Fig 1), survival and nursing physiology were only weakly affected by the different mixes and those changes were not linked to the pollen diet diversity. This suggests that among our pollen mixes other nutritional factors are more important than pollen species richness. For example, the “late July” mix consistently caused a significant reduction of longevity and nursing physiology. Those negative effects could be attributed neither to the presence of pesticide residues since this pollen mixture was the only one that was not contaminated (at a detection limit of 0.005 mg/kg), nor to its diversity index, which was above those of two others diets. Another explanation was that our pollen was stored seven years in freezer when we tested it, but a study of Dietz and Stevenson [63] showed that the nutritional value of fresh pollen frozen and stored in a freezer for one year and fresh pollen frozen and stored for 11 years were similar in regards to brood rearing. And all our mixes were stored 7 years. The “late July” mix was among the least consumed with “early May” and “early June”, but the last two mixes were those which cause the better survival to bees that have consumed them. However, this “late July” mix had the lowest protein and lipid contents, both of which are essential for worker health [16,64]. The “late July” period corresponded to the flowering of maize in the area where the pollen was harvested, as confirmed by the presence of 69.8% of maize pollen by volume in the mix (Fig 1). Maize produces pollen of poor nutritional quality as it is deficient in histidine (an essential amino acid for honey bees [65]), although the amount of other essential amino acids can be high [16,66]. The high prevalence of this pollen type in the “late July” mix likely accounts for the low quality of this diet. Jacobs [67] showed that consumption of maize pollen had a negative impact on the longevity of honey bee workers reared in cages. Similarly, Höcherl *et al*. [66] observed that bees fed with maize pollen show a reduction in brood rearing ability and lifespan. However, they did not find an effect on bee immunocompetence. As indicated by our results, the lower brood rearing ability [66] could be due to the poor HPGs development and *vitellogenin* expression. The availability of different floral resources is expected to compensate for the low quality of specific pollens [30]. However, the predominance of maize pollen in this collection period may not have allowed for such compensation phenomenon to take place here. It seems that despite its low nutritional quality, this pollen is very attractive for colonies during this period of late July because it is abundant and can be easily harvested [68].

The variability of nutritional resources in agricultural landscapes can expose honey bees to reduced pollen availability and/or deficiency in diet quality. As a consequence, such nutritional stresses can directly impact worker longevity and nursing physiology, and thereby nursing capacities, as observed in our laboratory conditions. If confirmed under field conditions, a reduction of brood rearing capacity combined with a shorter lifespan of adults workers could directly impact colony population [8,31–34], and potentially lead to a higher turn-over between cohorts of workers and a suboptimal brood rearing by malnourished nurses [69]. Providing bees with a greater diversity of floral resources, such as with nearby flower strips, fallow land, or semi-natural habitats, represents one way to overcome nutritional stress across the seasons [6].

## Supporting Information

S1 Table List of pesticides analysed in all pollen (limit of quantification of 0.01 mg/kg and limit of detection of 0.005 mg/k). (XLSX)

S2 Table Nutritional and palynological composition of the seasonal pollen mixes (pollen types and percentage of pollen grains by volume). (XLSX)
